# Development of a Geant4‐based independent patient dose validation system with an elaborate multileaf collimator simulation model

**DOI:** 10.1002/acm2.12530

**Published:** 2019-01-23

**Authors:** Hyun Joon Choi, Hyojun Park, Wook‐Geun Shin, Jung‐in Kim, Chul Hee Min

**Affiliations:** ^1^ Department of Radiation Convergence Engineering Yonsei University Wonju Republic of Korea; ^2^ Department of Radiation Oncology Seoul National University Hospital Seoul Republic of Korea; ^3^ Institute of Radiation Medicine Seoul National University Medical Research Center Seoul Republic of Korea; ^4^ Biomedical Research Institute Seoul National University Hospital Seoul Republic of Korea

**Keywords:** DICOM‐RT interface, dose, Geant4, IMRT, multileaf collimator, VMAT

## Abstract

Despite the improvements in the dose calculation models of the commercial treatment planning systems (TPS), their ability to accurately predict patient dose is still limited. One of the limitations is caused by the simplified model of the multileaf collimator (MLC). The aim of this study was to develop a Monte Carlo (MC) method‐based independent patient dose validation system with an elaborate MLC model for more accurate dose evaluation. Varian Clinac 2300 IX was simulated using Geant4 toolkits, after which MC commissioning with measurements was performed to validate the simulation model. A DICOM‐RT interface was developed to obtain the beam delivery conditions including the hundreds of MLC motions. Finally, the TPS dose distributions were compared with the MC dose distributions for water phantom cases and a patient case. Our results show that the TPS overestimated the absolute abutting leakage dose in the closed MLC field, with about 20% more of the maximum dose than that of the MC calculation. For water phantom cases, the dose distributions inside the target region were almost identical with the dose difference of less than 2%, while the dose near the edge of the target shows difference about 10% between Geant4 and TPS due to geometrical differences in MLC model. For the patient analysis, the Geant4 and TPS doses of all organs were matched well within 1.4% of the prescribed dose. However, for organs located in areas with high ratio of leaf pairs with distances less than 10 mm leaf pair (LP_(<10mm)_), the maximum dose of TPS was overestimated by about 3% of the prescribed dose. These dose comparison results demonstrate that our system for calculating the patient dose is quite accurate. Furthermore, if the MLC sequences in treatment plan have a large ratio of LP_(short)_, more than 3% dose difference in normal tissue could be seen.

## 1 INTRODUCTION

Volumetric modulated arc therapy (VMAT) and intensity modulated radiotherapy (IMRT) are techniques for treating cancer that utilize highly conformal dose distributions generated by multileaf collimator (MLC) motion. The conformity and uncertainty in dose delivery of VMAT/IMRT are sensitive to the structural details of the MLC; thus, accurate MLC modeling is very important for successful patient treatment and for reducing side effects.^1^ However, detailed modeling of the complex MLC geometry to reflect its precise dosimetric properties is challenging in commercial treatment planning systems (TPS).^2^ Molineu et al.^3^ reported the results of an analysis of a multi‐institutional IMRT clinical trial using the anthropomorphic head and neck IMRT phantom at the Radiological Physics Center. In this study 81.6% of the 1139 irradiations at 763 institutions passed the gamma analysis criteria of 7%/4 mm when the calculated and measured dose distributions were compared. The head and neck IMRT phantom was introduced in 2001 and 10 yr of data analysis showed that the phantom pass rate increased from 66% to 81.6%. Molineu et al.^3^ reported that a key factor of the increment of the phantom pass rate is the improvement in the modeling of MLC leaves in TPS. Nevertheless, TPS still uses the MLC leaves with flat ends to simplify the dose calculations and compensates the rounded leaf transmission by shifting the leaf positions.^4^ This shifting distance is called as a dosimetric leaf gap (DLG) and many trials have endeavored to find the optimal DLG, with the goal of minimizing uncertainty in the typical patient plan.^5^, ^6^, ^7^, ^8^, ^9^ However, variations in leaf end shape cause the dosimetric effect to vary due to the irregular shape and size of the resulting fields; therefore, each dosimetric effect should be verified individually.^7^ In the case of dynamic MLC, the dosimetric effect of the radiation transmitted and scattered from the rounded leaf ends can exceed 10% of the total dose.^10^, ^11^, ^12^ Even a 1% improvement in dose delivery precision has been reported to increase the cure rate for early stage tumors by 2%.^13^ Moreover, a 5% change in dose can result in 10–20% change in tumor control probability or up to 20–30% change in normal tissue complication rates if the prescribed dose falls along the steepest region of the dose‐effect curve.^14^

According to the guidelines in the AAPM Task Group 53 (1998) and 119 (2009) publications, two‐dimensional (2D) planar dosimetry measurements (e.g., film) are recommended for evaluating the accuracy of TPS. However, 2D planar dosimetry measurements are limited in that they can only detect inaccuracies within the selected plane of treatment volume or organ at risk.^6^, ^15^, ^16^ While three‐dimensional (3D) dosimetry techniques are available, these techniques require multiple large‐volume detectors such as a radiochromic plastic dosimeter and a polymer gel, in addition to scanners for verifying 3D dose distribution such as an MRI machine and an optical‐computed tomography (CT) scanner.^6^, ^17^, ^18^

**Figure 1:**
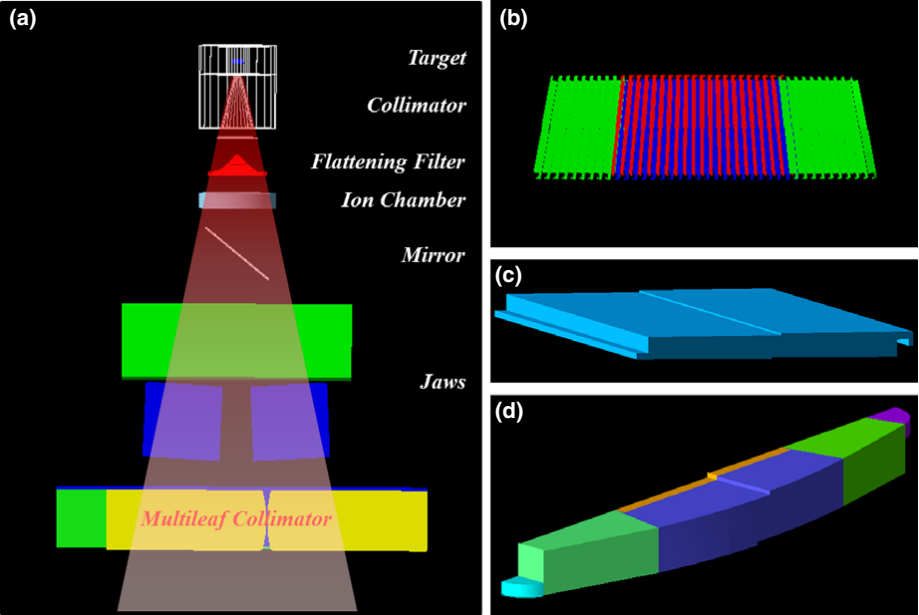
Geant4‐based MC modeling of (a) the Varian Clinac 2300 IX, (b) the 120‐leaf Millennium MLC, (c) leaf body, and (d) leaf rounded edge composed of four solid shapes.

The Monte Carlo (MC) method is considered the “gold standard” in assessing dose distribution and is one of the most appropriate methods for overcoming these limitations. This method has been applied to validate patient‐specific IMRT dose.^19^, ^20^, ^21^, ^22^, ^23^, ^24^, ^25^, ^26^, ^27^, ^28^, ^29^ EGSnrc/BEAMnrc is an optimal and efficient MC code for simulating linear accelerators (linacs) whose accuracy has been validated through many dosimetry studies.^30^ Accordingly, most studies aiming to develop a Monte Carlo‐based radiotherapy planning system are based on EGSnrc/BEAMnrc. However, DYNVMLC, which can model the geometry of the 120‐leaf Varian Millennium MLC in the BEAMnrc code, is designed to model the leaf end as a simple round shape.^31^ Since the actual shape of the leaf end of the Varian Millennium MLC is originally consisted of a circular arc at the center [Fig. 1(d)], two flat regions and two circular arcs with different radii and inner angle, dose evaluation error may occur due to incorrect leaf shape assumptions. In contrast, Geant4 is capable of sophisticated modeling of complex structures and is able to develop object‐oriented simulation systems that fit the user's purpose. Recently, Geant4 has shown excellent applicability to research fields such as the development of tetrahedral‐mesh‐based computational human phantoms and simulation of DNA strand damage.^32^, ^33^ In particular, Geant4 is able to handle dynamic geometry changes, which significantly facilitates true four‐dimensional Monte Carlo simulations, for example, dynamic MLC motion, patient organs, rotating machine parts, and moving scanners.^34^, ^35^ Although the calculation time of Geant4 is longer than that of EGSnrc/BEAMnrc, this obstacle could be overcome by increasing the computational power and implementing multithreading features. The aim of this study was to develop an independent dose calculation system for VMAT/IMRT with an automated DICOM‐RT interface and the linac head simulation using the Geant4 code. The MLC was modeled accurately for the evaluation of dose distribution from small fields and a moving MLC. The modeling of the linac head, including the MLC, was experimentally validated and the dose validation system was evaluated with three types of treatment plans.

## 2 MATERIALS AND METHODS

### 2.A Source modeling

#### 2.A.1 Linear accelerator modeling

A 6 MV Varian Clinac 2300 IX machine was modeled based on the manufacturer's information. Most of the major components of the linac head including the x‐ray target, primary collimator, beryllium window, flattening filter, ion chamber, mirror, jaws, and MLC were modeled in present work (Fig. 1). The Varian Millennium MLC was modeled using the Geant4 (version 10.00.p01) G4Tubs, G4Box, and G4ExtrudedSolid classes. Each leaf includes support rail, tongue, and groove geometry designed by the G4SubtractionSolid class. Each leaf was divided into two major sections, a rounded edge and a body. The rounded edge consisted of four solid shapes such as two quarter‐circles, one subtracted sector of a circle, two trapezoids, and/or one box. These solids were used to design a complex geometrical figure. In contrast, the body section was modeled simply by the G4ExtrudedSolid class. Ultimately, these solids were independently positioned in one mother volume. The Varian Millennium MLC consists of 60 leaf pairs (LPs) which have three types of leaf: full, half, and outboard. Each leaf design is mirrored on the opposite side of the bank. We defined x mm distance between paired opposite leaf tips as LP_(x mm)_ in this study.

#### 2.A.2 Beam commissioning

For the time‐consuming process of MC commissioning, variance reduction techniques were employed with bremsstrahlung splitting, Russian roulette, and generation of phase‐space (phsp) files. The bremsstrahlung splitting factor was 100 in the target and the Russian roulette factors were 0.01 in the primary collimator and jaws. Phsp files were recorded for incoming particles to the very thin layer upstream of the MLC. Three sets (30 × 30, 10 × 10, and 4 × 4 cm^2^) of percent depth dose (PDD) data and lateral profiles at depths of 5 and 10 cm were compared between the Geant4 and the Golden Beam Data (GBD) provided by the manufacturer (Varian Medical Systems, Palo Alto, CA) to tune the characteristics of the initial electron beam.^36^, ^37^, ^38^, ^39^ The dimensions of the water phantom (e.g., blue phantom^2^ scanning volume) were 48 × 48 × 41 cm^3^ and the PDD and lateral dose distributions were calculated with 2 × 2 × 2 mm^3^ voxels.

### 2.B Experimental validation of the modeled MLC

**Figure 2:**
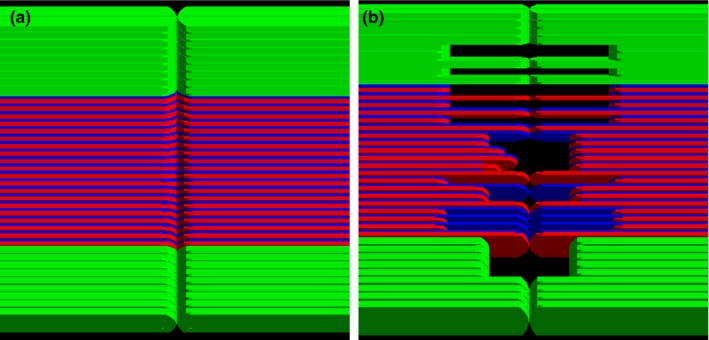
Two kinds of beam field for the film measurements made to validate the MLC model; (a) closed MLC field, (b) in‐house designed MLC field.

For evaluation of the modeled MLC, two kinds of film measurement were performed with a solid water phantom using a Varian Clinac 2300 IX instrument (Fig. 2):

1. Transmission of a 10 × 10 cm^2^ beam through the closed MLC field to evaluate the interleaf and abutting leakage doses at a source‐to‐detector distance (SDD) of 100 cm and
2. Transmission of a 20 × 30 cm^2^ beam through an in‐house MLC field to assess beam divergence and relative output at an SDD of 100 cm.

For each measurement, 400 monitor units (MUs) were delivered to Gafchromic EBT2 (International Specialty Products, Wayne, NJ, USA) film placed at 1.5 and 5 cm depth along the beam direction for the first and second measurement, respectively. Films were scanned using an Epson expression 10000XL scanner (Epson America Inc., Long beach, CA, USA). This scanner allows the acquisition of red‐green‐blue (RGB) transmission images from the film. RIT (Radiological Imaging Technology, CO, USA) software was used to analyze the transmission images. Dose response calibration of EBT2 films was performed using the procedure provided by the manufacturer. The relative transmission values for the film measurement were compared to those for the Geant4 and TPS. To calculate the dose distributions, voxelized water phantoms with 0.93 × 0.93 × 1.50 mm^3^ and 1.00 × 1.00 × 1.00 mm^3^ voxels were used for the Geant4 and TPS, respectively.

### 2.C Development of the DICOM‐RT interface

**Figure 3:**
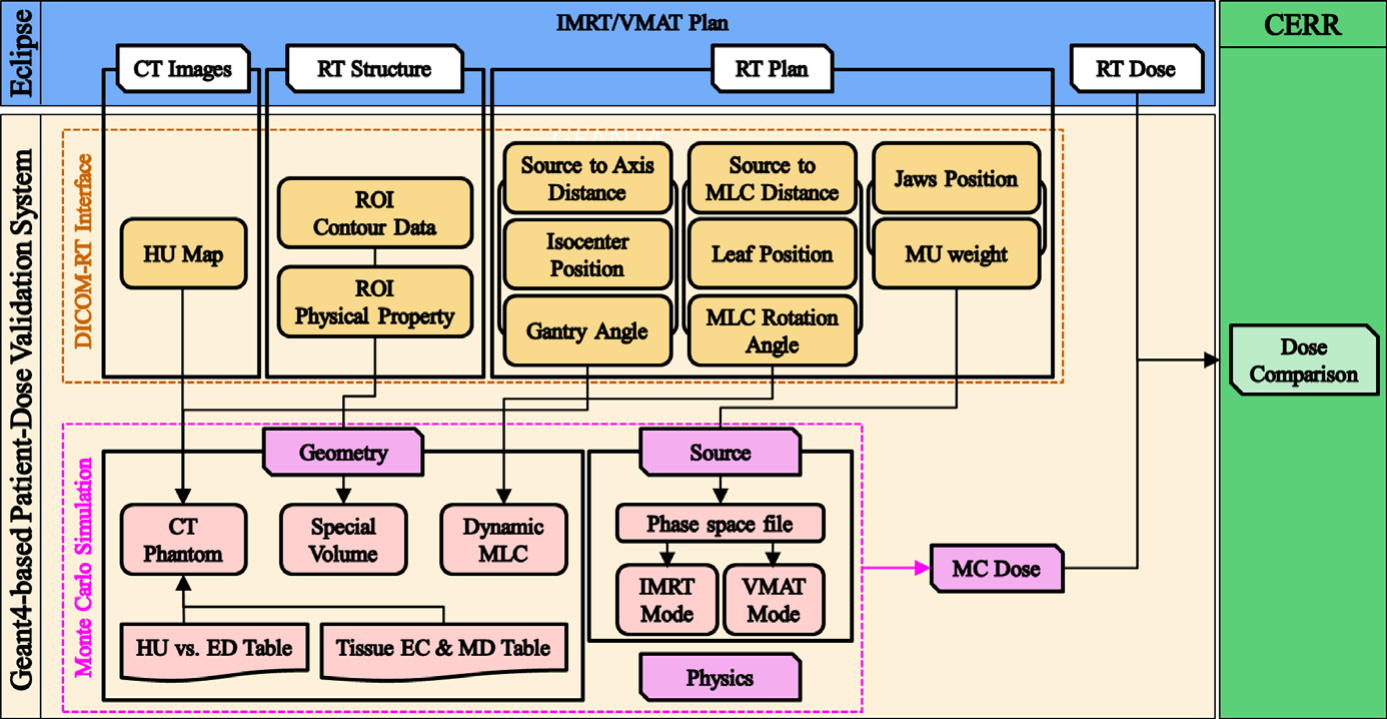
Flow chart of the in‐house patient dose validation system.

For MC simulations of radiotherapy plans, the automated DICOM‐RT interface is essential due to many beam delivery parameters in the treatment plans. The DICOM files for radiotherapy planning consist of four file types: CT images, RT structures, RT plans, and RT doses. These files have a format for storing information associated with a value representation (VR) that indicates the encoding type and a tag that uses 8‐digit hexadecimal numbers.^40^ To extract patient‐dependent parameters, data can be discriminated using the DICOM tag while reading the file. The DICOM file reading process was benchmarked against the Geant4 example. A flow chart of the in‐house patient dose validation system, including the automated DICOM‐RT interface, is illustrated in Fig. 3. In this system, DICOM files are created from the Eclipse^™^ TPS for patient‐specific VMAT/IMRT planning and the DICOM‐RT interface extracts the patient‐specific parameters needed for the Monte Carlo simulation from each file.

The dose validation system extracts dimension information from CT images and the HU value of each pixel and then models the geometry of the CT images using the G4PVReplica class. Next, material is assigned to each pixel with the HU to electron density (ED) conversion lookup table. While the table is made from CT images of the ED phantom (Model 062M, Computerized Imaging Reference Systems, Norfolk, VA, USA), the elemental composition (EC) and mass density (MD) of each material are not provided in this table. Kim et al.^41^ reported that Geant4‐based MC dose distributions can be significantly affected by the material conversion method. Therefore, the Schneider material conversion method was used for patient cases.^42^ If a special volume (e.g., the fiducial marker, virtual water phantom, and couch) is present in the CT image, EC and MD would be defined in the region of interest (ROI) based on the physical property value stored in RT structure file; this process was automated in our system.

The patient‐dependent parameters extracted from the RT files were stored as editable text files. The modeled patient phantom was rotated on the isocenter instead of the linac head including many components in it. Dynamic MLC movement was determined by the sequence of leaf positions for each control point. The leaf positions stored in the RT plan file are the positions at the isocenter. Since the leaf positions do not consider beam divergence, the physical leaf position in the simulation was assessed considering the beam divergence as the ratio between the source to the MLC and the source‐axis distance. The horizontal MLC rotation angle was considered to minimize the leakage dose from the MLC. The total number of particles is related to the total MU determined in TPS and the number of particles for each control point was determined according to the MU weight information. The MU weight is the same as the segment MU divided by the total MU. However, the number of particles for each control point is differently calculated between IMRT and VMAT. For IMRT simulations, the number of particles is zero for the first control point and the dose calculation starts from the second control point according to the MU weight. On the other hand, for VMAT simulation, only half MU weight for the first and end control points was applied to calculate the number of particles, otherwise, the average MU weight for the current and previous control points was applied. IMRT/VMAT simulation was performed with electromagnetic process in G4EmStandardPhysics_option3.

Finally, the Geant4 dose distribution was compared with the TPS dose distribution using Computational Environment for Radiation Research (CERR) software.^43^ The CERR program allows users to handle DICOM files in Matlab and analyze dose distributions with dose‐volume histograms (DVH) and 3D dose differences.^43^

### 2.D Evaluation of VMAT/IMRT plans

The VMAT/IMRT plans calculated by the TPS (Eclipse^™^ version 8.9) using the Analytical Anisotropic Algorithm (AAA) were compared with the plans calculated by the in‐house patient dose validation system. In the VMAT/IMRT plan, the DLG value and the leaf transmission were determined to be 0.165 cm and 1.7%, respectively. In order to predict the absolute dose in a subject, a dose scaling factor (DSF) needed to be calculated because we cannot use the same amount of primary electrons as that used in the real treatment for the Geant4 simulation. We determined the DSF by finding a relationship between the dose calculated by the TPS with 100 MU and the dose calculated by the Geant4 with 2 × 10^9^ primary electrons in the water phantom. The reason for using 2 × 10^9^ primary electrons in Geant4 to determine the DSF was that the statistical error of the dose distribution was less than 2% and it was small enough to decide the maximum dose in the water phantom. The dose distribution in the water phantom was calculated by using 10 × 10 cm^2^ formed with only jaws. After that, the DSF was calculated by matching the maximum dose value in the central depth dose distributions calculated by the Geant4 to that calculated by the TPS. Whenever applying this DSF to the dose distribution of different treatment plan, the DSF should be divided by the ratio of the used number of primary electrons and 2 × 10^9^ and multiplied by the MU set in the TPS. To evaluate an absolute dose difference caused by different leaf end shape on both closed and open region of the MLC at a time, the dose distribution in the water phantom was calculated with a photon beam formed by different field sizes of MLC and jaws—which were 10 × 10 cm^2^ and 15 × 15 cm^2^, respectively. Furthermore, three treatment plans were created for VMAT of a patient case and VMAT/IMRT of a water phantom case. The HU values outside of the patient's body contour were manually assigned as −1000 to eliminate the dose calculation difference due to noise. The dose grid sizes for the TPS were 1.00 × 1.00 × 1.00 mm^3^ and 2.00 × 2.00 × 2.00 mm^3^ in the water phantom and the patient cases, respectively. To assess treatment quality in the target volume, four dose‐volumetric parameters such as dose received by at least 95% of the volume (D_95%_), mean dose (D_mean_), near minimum dose (D_98%_), and near maximum dose (D_2%_) were calculated. For the normal organs, D_mean_ and D_2%_ were evaluated.

## 3 RESULTS AND DISCUSSION

### 3.A Beam commissioning

**Figure 4:**
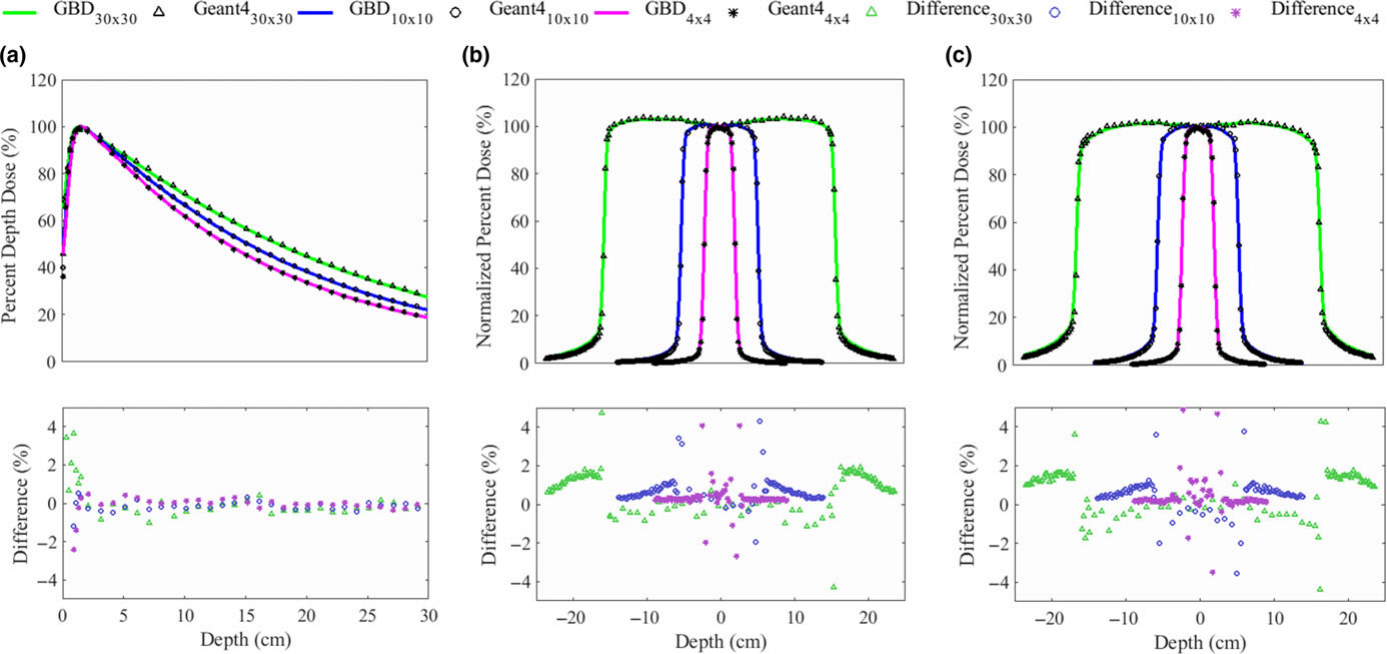
MC commissioning by comparing the measurement and MC results; (a) PDD profiles, (b) lateral dose profiles at a depth of 5 cm, (c) lateral dose profiles at a depth of 10 cm.

For the first validation process of the independent patient dose calculation system, the MC commissioning was performed by comparing the GBD and the MC simulations. The MC commissioning determines four characteristics of the initial electron beam. The mean energy, standard deviation of Gaussian energy distribution, beam radius, and standard deviation of Gaussian radial distribution were 5.9 MeV, 0.83 MeV (FWHM 33% of mean energy), 0, and 1.06 mm (FWHM 2.5 mm), respectively.^39^ Figure 4 shows the MC commissioning results for field sizes of 4 × 4, 10 × 10, and 30 × 30 cm^2^. The dose differences between the GBD and Geant4 were evaluated for all range of distribution except for the steep dose fall‐off region. The reason for this exception will be discussed in the next section. The PDD profiles calculated by the Geant4 matched well with the GBD. Specifically, the local differences of each other were within 1.5% for all field sizes.^39^ The lateral dose profiles calculated by the Geant4 for all field sizes agreed with the GBD within 1.6% including the outer penumbra region.^39^ The statistical fluctuations in computed dose distributions were about ±0.6%. The calculations were performed on two Intel Xeon E5‐2697V2 CPUs (12 cores) at 2.7 GHz and two Intel Xeon E5‐2697V3 CPUs (14 cores) at 2.6 GHz. The phsp files were recorded for each field with 5.6 × 10^8^ primary electrons; the calculations took about 396, 449, and 207 CPU‐hours for the 4 × 4, 10 × 10, and 30 × 30 cm^2^ fields, respectively. To calculate the PDD and profiles, the recorded phsp files were reused 60, 70, and 70 times. The simulation times were about 39.6, 270, and 860 CPU‐hours for the 4x4, 10 × 10, and 30 × 30 cm^2^ fields, respectively.^39^

### 3.B Experimental validation of the MLC simulation model

**Figure 5:**
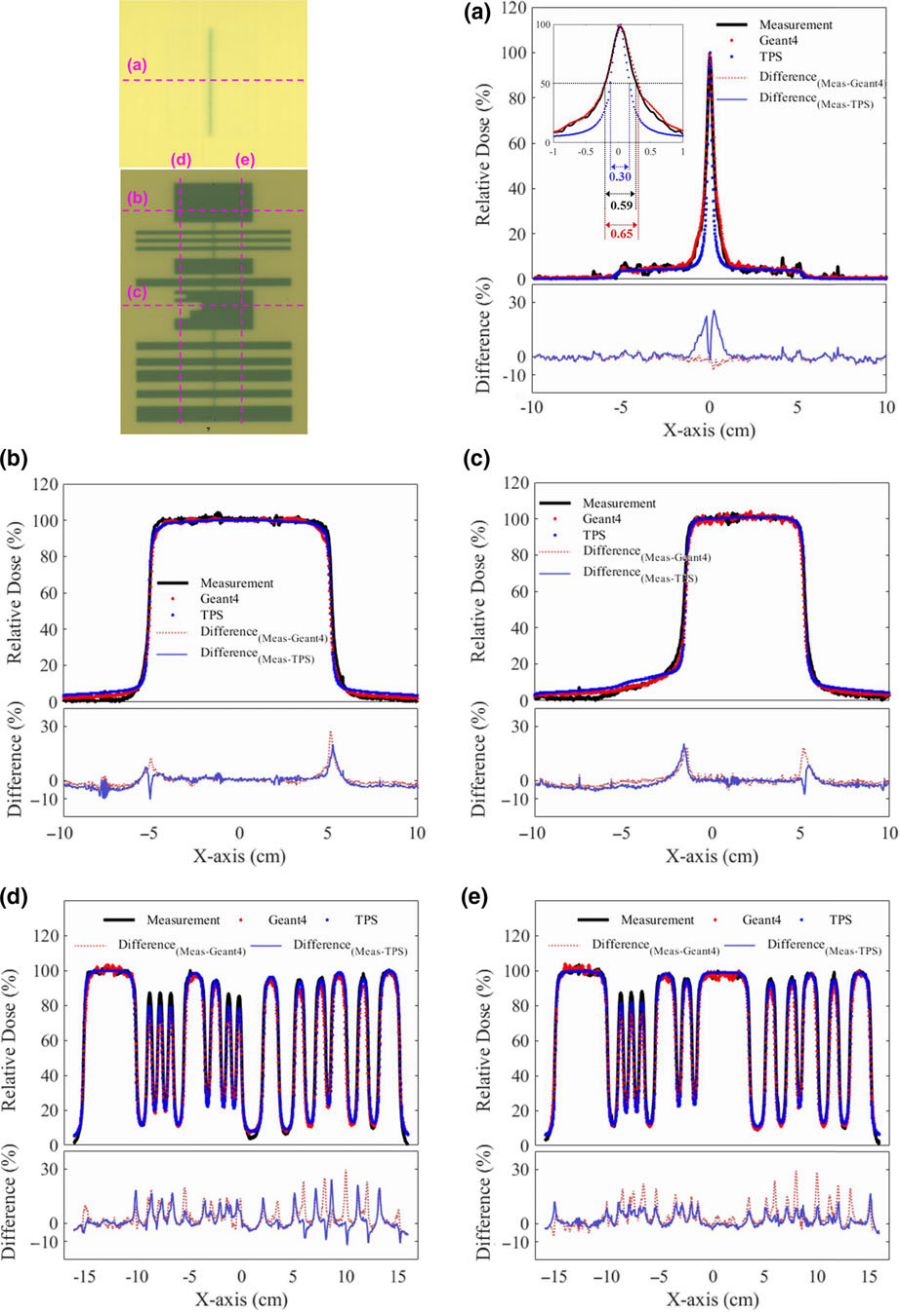
Comparison of dose distributions achieved with the closed MLC field and the in‐house MLC field; Central x‐direction profile for the dose distribution of the closed MLC field (a), two x‐direction profiles for dose distribution of the in‐house MLC field (b)‐(c), two y‐direction profiles for dose distribution of the in‐house MLC field (d)‐(e).

The second validation process of the in‐house system was film measurement to validate the geometrical modeling of MLC. Figure 5 shows the measured and calculated dose distributions in two different MLC conditions. The fluctuation of transmission values, the spatial resolution uncertainty, and the nonuniformity of film measurements were about ±2.4%, 0.08 mm, and 0.2%, respectively. The statistical fluctuations in the dose distributions computed using Geant4 were about ±2.5%. The calculated and measured dose distributions were matched well with each other. To assess dose distributions, the central x‐direction profile [Fig. 5(a)] of the closed MLC field and the four x and y‐direction profiles [Figs. 5(b)–5(e)] of the in‐house MLC field were compared. Graphs (a) of Fig. 5 shows the dose comparison of the interleaf and abutting leakage. The interleaf leakage dose was estimated by calculating the average relative dose in the field of the jaws except for the peak area. The interleaf leakage dose of the three dose distributions agreed within 1% of each other. The abutting leakage dose corresponding to the peak region of the profile was assessed by comparing the full width at half maximum (FWHM) of the peaks. The FWHMs for the Geant4, TPS, and experimental measurement were 0.65, 0.30, and 0.59 cm, respectively. These results show that the TPS peak is sharper than the experimental measurement and Geant4 peaks, which implies that the TPS does not properly reflect the abutting leakage dose with the closed field, while that of the measurement and MC were almost identical. In the dose distribution of the complex MLC field, the four x‐ and y‐direction profiles [Figs. 5(b)‐5(e)] show the results of the assessment of x‐ and y‐direction beam divergence. The four profiles of the Geant4, TPS, and measurement were matched well with each other, even though there are some differences at dose fall‐off regions. These dose differences could be caused by the difference of the grid size which could significantly effect on the dose calculation especially at steep dose fall‐off region. Note that, the grid sizes in x‐ and y‐direction for the Geant4, TPS, and measurement were 0.93 and 1.50 mm, 1.00 and 1.00 mm, and 0.17 and 0.17 mm, respectively. Furthermore, the position of dose calculation in the depth direction can be another factor because the photon beam is divergent to the depth direction and scattered in the water phantom. Compared to the closed field, the diverging characteristics of the photon beam are noticeable in the opened MLC field. The dose of the TPS in the outer penumbra region of the x‐direction profiles was overestimated than that of the Geant4 and measurement because of the 1.7% leaf transmission set in TPS. We assumed that the reason for this overestimation is to compensate dosimetric difference at dose fall‐off region caused by the flat‐end shape of MLC.^44^ The second validation process demonstrated the accuracy of the current MC simulation with film dose measurements for two kinds of MLC field.

**Figure 6:**
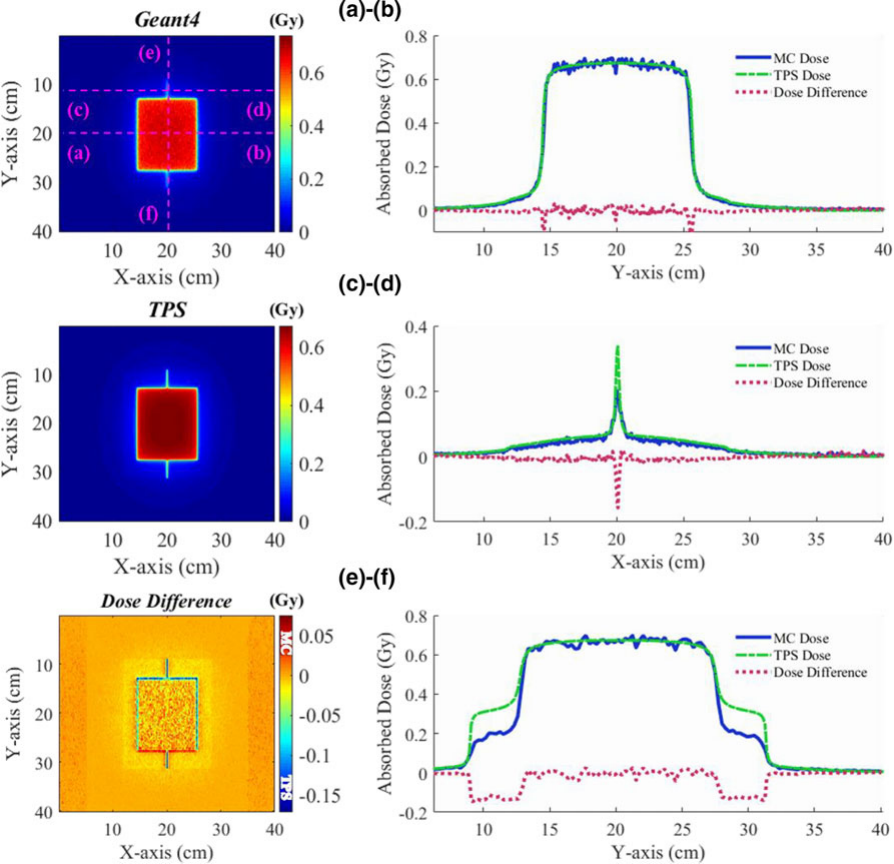
Dose distribution in the MLC‐defined 10 × 10 cm^2^ field (jaws at 15 × 15 cm^2^); Central x‐direction dose profile (a), upper x‐direction dose profile in the close MLC field (b), central y‐direction dose profile (c).

Figure 6 shows the dose distribution in the MLC‐defined 10 × 10 cm^2^ field (jaws at 15 × 15 cm^2^). The statistical fluctuation in the dose distribution computed using the Geant4 was about ±1.2%. The abutting leakage dose and the slope of the dose fall‐off region differ between the Geant4 and the TPS. The dose profile of (a) in Fig. 6 shows about 0.1 Gy difference (about 16% of the maximum dose) at the dose fall‐off region, this difference could be due to the assumption of the DLG value in TPS. Furthermore, the profiles (b) and (c) indicate that the TPS overestimates the absolute dose of abutting leakage, up to 20% of the maximum dose than the dose assessed by the Geant4. As discussed in Fig. 5, the overestimation of the dose of the TPS in the outer penumbra region was also observed in Fig. 6 and it was about 1.5% of the maximum dose. This means that if the ratio of the LPs_(short)_ is dominant in the complex VMAT/IMRT plan, the dose in the normal organs or tissues could be overestimated. Moreover, the dose difference map in Fig. 6 shows that the dose at the edges of the field in the y‐direction differs by about 7% of the maximum dose.

### 3.C Evaluation of the VMAT/IMRT plan

#### 3.C.1 Water phantom cases

**Figure 7:**
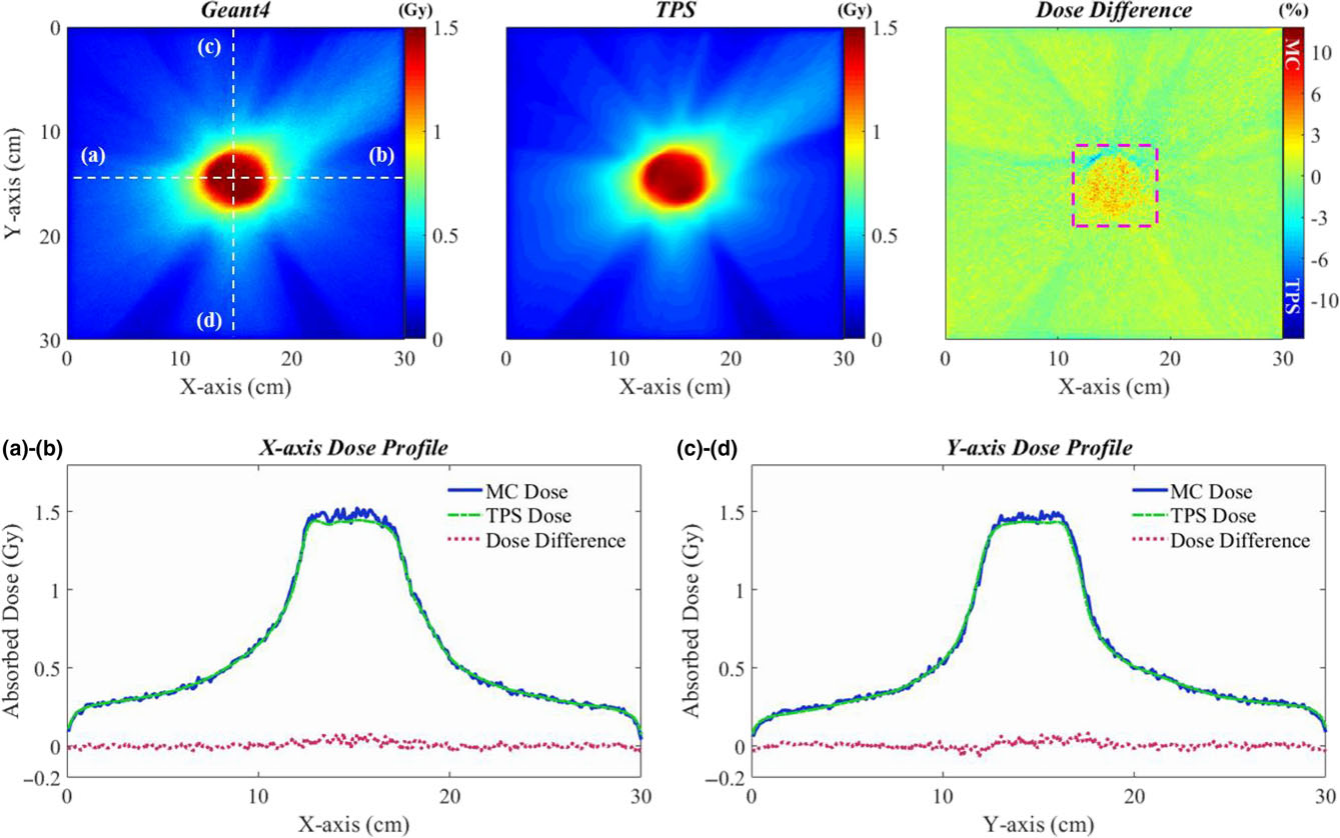
Comparison of dose distributions and central dose profiles of the VMAT plan in water with those calculated by Geant4 and the TPS; Central x‐direction profile of the isocenter‐plane dose distribution (a), central y‐direction profile of the isocenter‐plane dose distribution (b).

**Figure 8:**
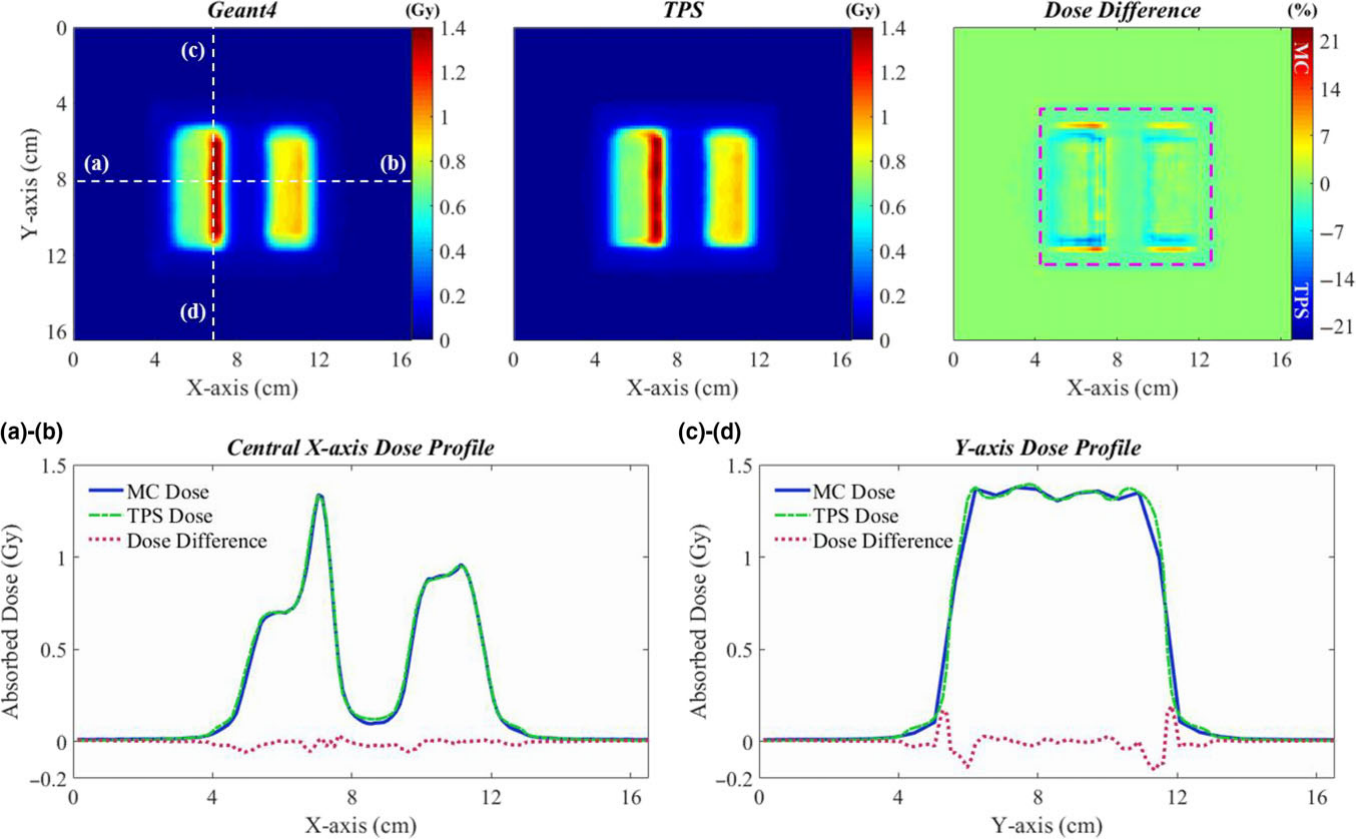
Comparison of dose distributions and central dose profiles of the unidirectional IMRT plan in water calculated by Geant4 and the TPS; Central x‐direction profile of the isocenter‐plane dose distribution (a), y‐direction profile of the isocenter‐plane dose distribution (b).

The final validation step of the Geant4‐based patient dose calculation system was its application to clinical VMAT/IMRT cases with the DICOM‐RT interface. Figures 7 and 8 show the comparison of the VMAT and IMRT dose distributions calculated by the Geant4 and TPS in water. Dose differences were assessed via pixel‐by‐pixel comparison. In the VMAT plan, the 6 MV photon beam was delivered to the isocenter with 160 different gantry angles and their control points. To disperse the overlapped MLC leakage dose with the opposite directions, the MLC was horizontally rotated by 10 degrees. The maximum dose and the dose at the isocenter were 1.59 and 1.43 Gy, respectively, with 312.5 MU in the TPS. The statistical fluctuation in the dose distribution computed using Geant4 was about ±1.1% in the target volume. For the patient dose calculation, 5.6 × 10^9^ initial electrons were used and the simulation time was about 588 CPU‐hours. As shown in Fig. 7, the Geant4 and TPS central dose distributions matched quite well with each other. The average and maximum dose differences in a box‐shaped ROI (pink dotted line) on the dose difference map in Fig. 7 were 1.5% and 14.4%, respectively.

In the IMRT plan, the 6 MV photon beam was delivered in one direction with 160 control points. With the TPS, 191 MUs were delivered; the maximum dose and the dose at the isocenter were 1.39 and 0.05 Gy, respectively. The statistical fluctuation in the dose distribution computed using Geant4 was about ±0.1% in the target area at the depth of maximum dose. An initial electron beam of 1.12 × 10^10^ particles was used in the MC simulation with 952 CPU‐hours. In this plan, the mean and maximum dose differences between the Geant4 and TPS were 2.0% and 13.8% in a box‐shaped ROI of the XY‐plane (Fig. 8). Dose difference in y‐direction dose profile (b) results from different slope angles of dose fall‐off region and different simulation models of tongue‐and‐groove for shielding interleaf leakage radiations could be a reason for the different slope angles.

**Figure 9:**
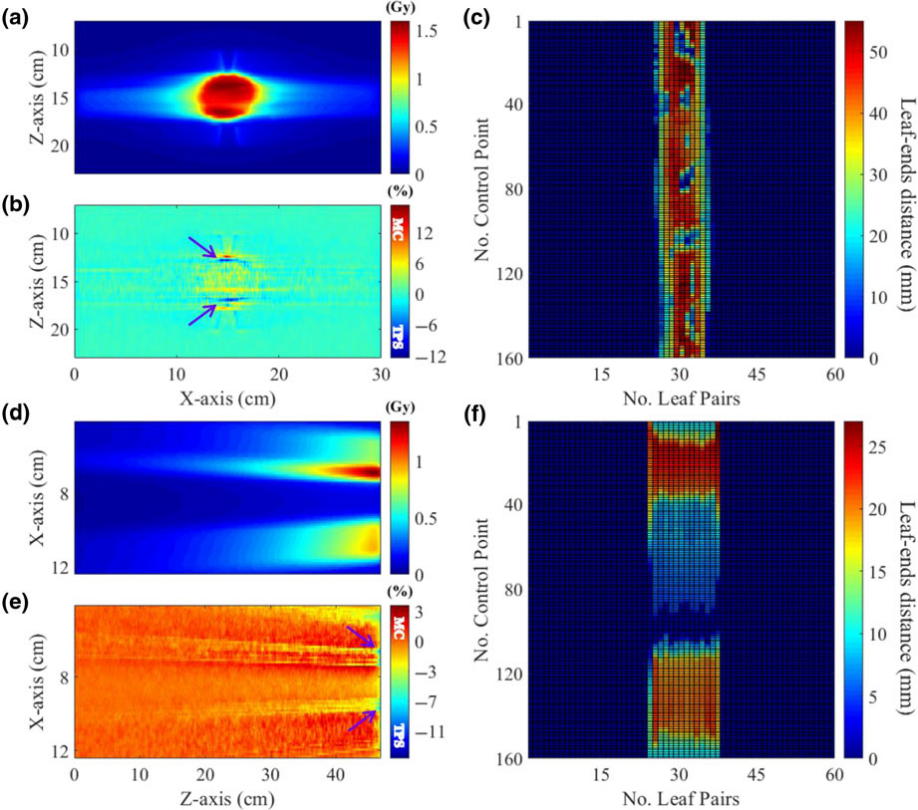
The VMAT/IMRT dose distributions in the XZ‐plane [(a) VMAT, (d) IMRT], the dose difference maps with purple arrows indicating the high TPS dose region [(b) VMAT, (e) IMRT], and the map of leaf‐ends distance according to control point of the VMAT/IMRT plan in water [(c) VMAT, (f) IMRT].

As found in the validation process of MLC simulation model, more than 10% dose difference was found in some local areas of VMAT/IMRT dose distribution. The dose difference could be due to the difference in the dose grid size that is quite sensitive to the LPs_(short)_ of the MLC movement plan. Figure 9 shows the VMAT/IMRT dose distributions in the XZ‐plane [(a) VMAT, (d) IMRT] and the dose difference maps [(b)VMAT, (e) IMRT], and the leaf‐ends distance maps [(c) VMAT, (f) IMRT] according to control points of the treatment plan. The dose grid size for the Geant4 in x‐ and z‐direction of Figs. 9(b) and 9(e) were 0.78 and 5.00 mm, and 1.17 and 5.00 mm, respectively. In Fig. 9(a), the V‐shaped dose distributions on the outside of the target volume are the abutting leakage dose distributions resulting from the 360 degrees gantry rotation and we could observe in Fig. 9(b) that TPS overestimated the dose about 3% in that region. The dose distributions in the regions indicated by the purple arrows in Fig. 9(b) are caused by the motion of 25th and 35th LPs of the total 60 LPs [Fig. 9(c)]. As shown in the leaf‐ends distance map for the VMAT plan, the 25th and 35th LPs are mostly LPs_(<10mm)_ over the 160 control points. The dose differences in that regions are about 10%, which could be caused by the accumulated dose from LPs_(<5mm)_ because the dose grid size for the TPS was 1.00 × 1.00 × 1.00 mm^3^. The IMRT dose distribution [Fig. 9(d)] is made by accumulating the dose with 160 control points moving from the top to the bottom of the XZ‐plane with the MLC movement plan illustrated in Fig. 9(f). In the dose difference map [Fig. 9(e)] showing the yellow, orange, and red linear patterns, the every color except red patterns indicates higher TPS dose than Geant4 dose. The pattern of LPs_(<10mm)_ of the map for the IMRT plan is almost consistent with the orange and yellow patterns ranging from 0 to 3% dose difference and these patterns could be caused by the absolute dose difference resulting from LPs_(<10mm)_. The dose differences at the region indicated by the purple arrows are over 7%. We assumed that the high dose difference only near the surface of the water phantom could be caused by the difference in the dose grid sizes which for the Geant4 and the TPS were 1.17 and 1.00 mm, respectively. As the depth of the water phantom becomes deeper, it is presumed that the dose difference is reduced due to the phantom scatter. However, despite these local dose differences, the dose comparison study in the water phantom indicates almost identical dose distributions between the Geant4 and TPS.

#### 3.C.2 Patient case

**Figure 10:**
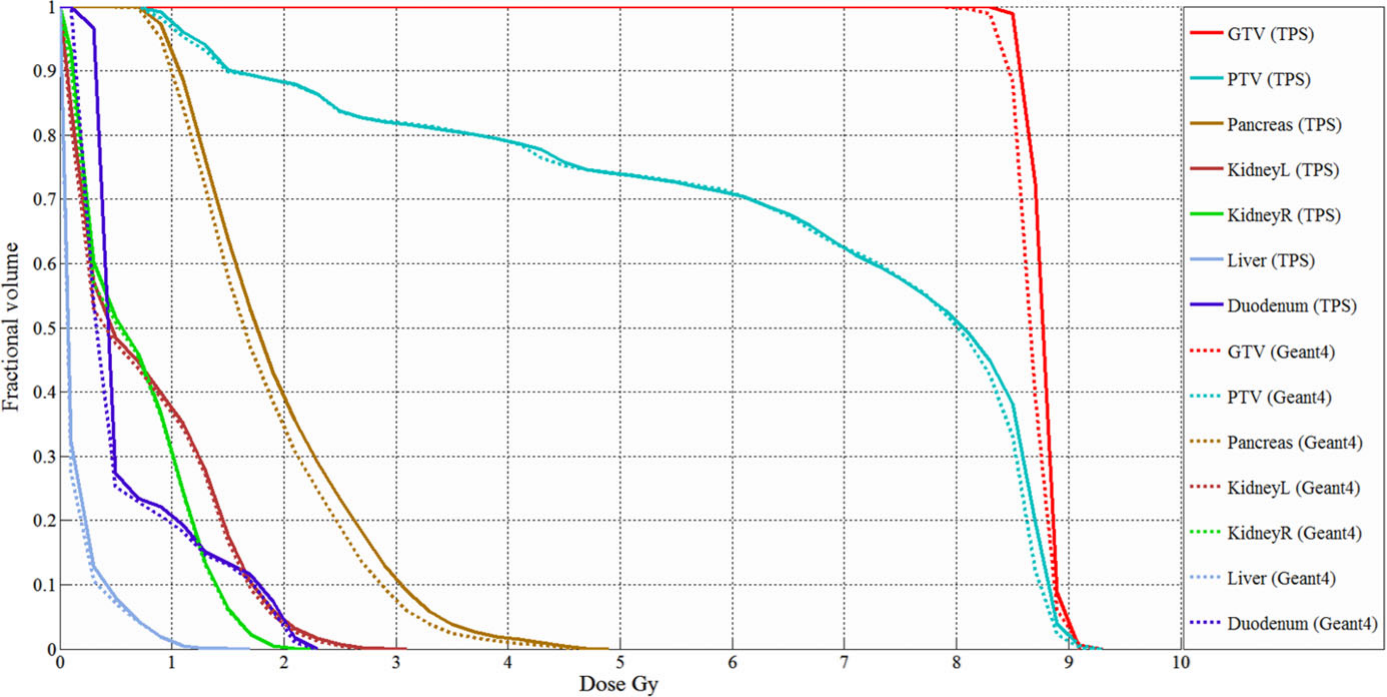
Dose volume histogram of the abdomen case with Geant4 and the TPS.

Figure 10 and Table 1 show the DVH of the abdomen case and dose volumetric parameter values of the target volumes and normal organs, respectively. The dose differences in Table 1 were calculated based on the prescribed dose. The 6 MV photon beam was delivered to the isocenter with 178 gantry angles in two opposite directions. To distribute the leakage dose, the MLC was horizontally rotated by 10 and 350 degrees for each gantry rotation direction. The doses at the isocenter were 5.25 and 3.49 Gy with 1103.6 and 1085.7 MUs for the two opposite beams, respectively. The statistical fluctuations of the dose calculated using the Geant4 to each voxel in normal tissue and target regions were about ±1.1% and ±0.3%. For the Geant4 calculations, 5.6 × 10^9^ initial electrons were used and the simulation took 336 CPU‐hours for each gantry rotation direction. The abdomen CT images consist of 22 structures. Major organs such as the pancreas, kidney, and duodenum were selected for dose assessment. The dose grid size for the Geant4 was 1.27 × 1.27 × 2.00 mm^3^ and as observed in the water phantom study, the dose difference occurred at the edge of the target volume, while the other regions were almost identical between the Geant4 and TPS calculations. For the planning target volume (PTV) and gross tumor volume (GTV) volume, the dose‐volumetric parameters were within 1% and 2.2% each other, respectively (Table 1). With the exception of the pancreas, the normal organs were in good agreement within 1.1% for all dose parameters (Table 1). However, D_2%_ in the pancreas calculated by the TPS was overestimated by 3% in comparison with the Geant4 calculation (Table 1).

**Table 1:** Dose‐volumetric parameters of target volumes and normal organs for the abdomen case

	GTV (Gy)				PTV (Gy)			
	D_95%_	D_mean_	D_98%_	D_2%_	D_95%_	D_mean_	D_98%_	D_2%_
TPS	8.52	8.86	8.50	9.05	1.20	6.71	0.96	9.01
MC	8.37	8.76	8.31	9.04	1.12	6.66	0.91	8.92
Dose difference^a^	1.7%	1.1%	2.2%	0.1%	0.9%	0.6%	0.6%	1.0%

	Kidney L (Gy)		Kidney R (Gy)		Pancreas (Gy)		Duodenum (Gy)	
	D_mean_	D_2%_	D_mean_	D_2%_	D_mean_	D_2%_	D_mean_	D_2%_
TPS	0.85	2.24	0.76	1.73	2.03	3.88	0.77	2.09
MC	0.82	2.18	0.75	1.72	1.91	3.62	0.67	2.05
Dose difference^a^	0.3%	0.7%	0.1%	0.1%	1.4%	3.0%	1.1%	0.5%

a Dose differences are based on the prescribed dose (8.74 Gy).

**Figure 11:**
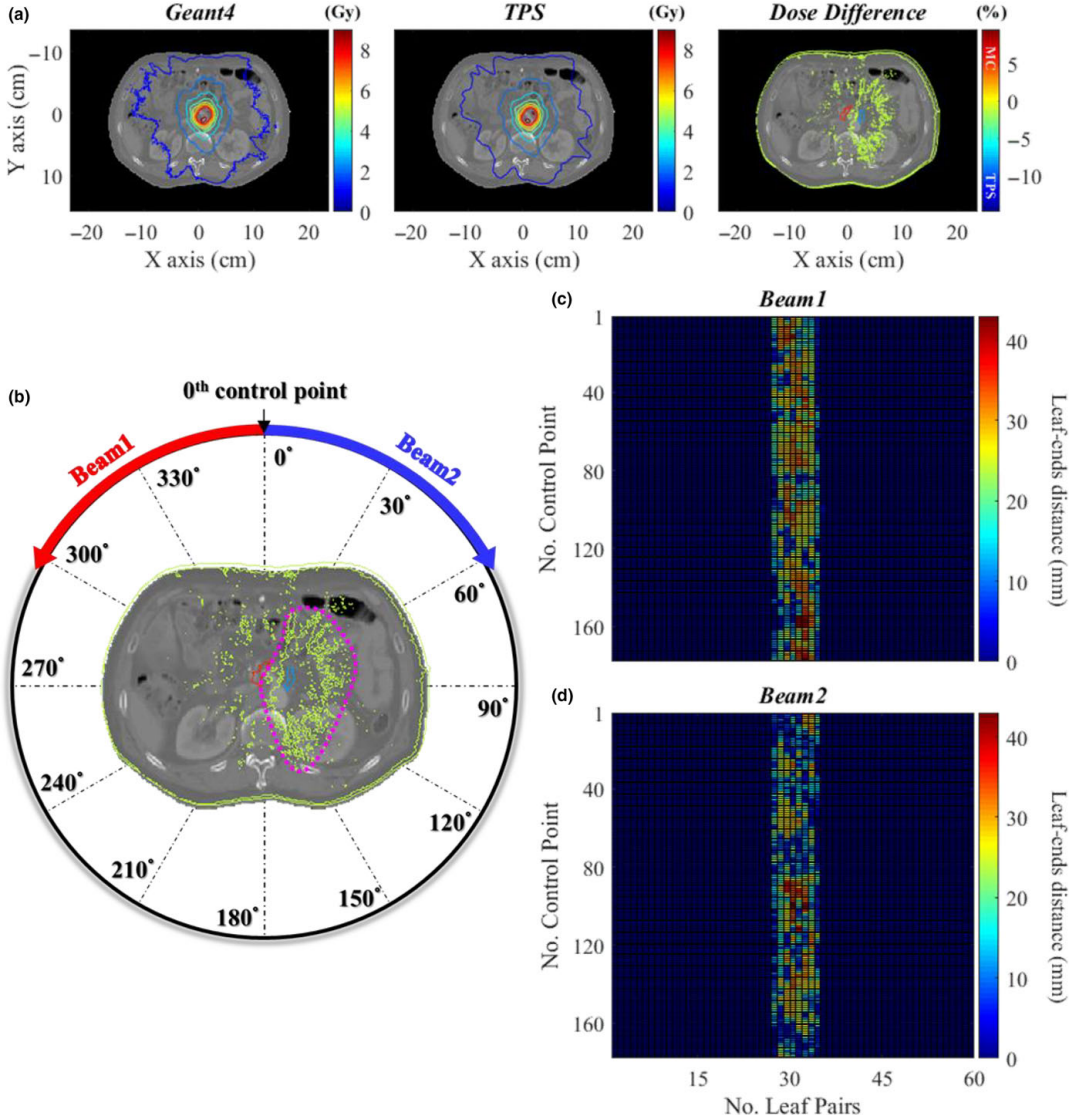
Comparison of dose distributions of the VMAT plan for the abdomen case calculated by Geant4 and the TPS [(a)], description of therapeutic beam delivery process according to control point of the VMAT plan for the patient [(b)], the maps of leaf‐ends distance according to control point of the VMAT plan [(c) Beam1, (d) Beam2].

The patient dose distributions and the two leaf‐ends distance maps for the patient case in which the two beams are rotated 360 degrees in opposite directions are illustrated in Fig. 11. Beam 2 has a larger ratio of LPs_(short)_ than beam 1. In the map of beam 2, the ratio of LPs_(short)_ at control points between 1 and 80 is higher than that from 81 to 178. Since the gantry rotates about 2 degrees for a control point, the total rotation angle can be assumed as 160 degrees from the first to the 80th control point. In the dose difference maps, green and blue contours indicate higher dose of TPS than that of Geant4 and especially the higher dose of TPS is noticed with the fan‐shaped distribution (pink dotted line) on the right side of the XY‐plane. We observed a relationship between the higher dose distributions of TPS and the distributions of LPs_(short)_. Because the pancreas was placed in the region of higher TPS dose, there was 3% difference of D_2%_. Moreover, the differences of four PTV dose‐volumetric parameters between Geant4 and TPS were about 1% or less, whereas the relative differences of the GTV (smaller volume than PTV) parameters were as high as 2.2%. We assumed that if a volume of organ or tissue of interest is very small, the dose‐volumetric parameters of corresponding organ or tissue could be sensitive to the local dose differences caused by the LPs_(short)_.

## 4 CONCLUSION

In this study we developed a Geant4‐based independent patient dose validation system including a finely modeled MLC and automated DICOM‐RT interface. The developed system was validated by three processes: MC commissioning of the modeled linac, experimental validation of the modeled MLC, and dose comparison in water between the commercial TPS and Geant4. Finally, the patient dose distribution calculated by the TPS for an abdomen case of the VMAT plan was evaluated using developed MC system. As a result of the validation, it was confirmed that the in‐house MC system was able to accurately evaluate the patient dose sufficiently. However, we found that the rounded leaf end of MLC could cause the dose difference compared to the TPS in the case of LPs_(<10mm)_. Da Rosa et al.^45^ investigated the influence of lung heterogeneity on dose distribution in a soft tissue phantom. They evaluated PDD curves in the phantom by comparing between the dose calculated by MC method, by TPS with four algorithms, and experimental data according to the different field size from 1 × 1 cm^2^ to 10 × 10 cm^2^.^44^ As the results of this study, the dose difference was increased up to about 40% in the region of lung‐tissue equivalent material comparing between MC and AAA for the 1 × 1 cm^2^ field due to the lateral electronic disequilibrium effect.^44^ In our results, about 3% difference of the prescribed dose in the normal tissue could cause by a large ratio of LPs_(<10mm)_ in the treatment plan, even though the patient case is not that heterogeneous case. In other words, the effect of the short leaf‐ends distance in a highly heterogeneous region can result in a significant dose difference. Therefore, it is necessary to quantitatively analyze the correlation between the ratio of LPs_(short)_ and the dose difference. In the future, several treatment plans for highly heterogeneous media (e.g., lung case, head & neck case, and dummy shield case) will be evaluated with the currently developed dose validation system.

## CONFLICT OF INTEREST

No conflicts of interest.
